# Contemporary treatment utilization among women diagnosed with symptomatic uterine fibroids in the United States

**DOI:** 10.1186/s12905-020-01005-6

**Published:** 2020-08-13

**Authors:** Nicole Gidaya Bonine, Erika Banks, Amanda Harrington, Anna Vlahiotis, Laura Moore-Schiltz, Patrick Gillard

**Affiliations:** 1 Health Economics & Outcomes Research – Canada, Allergan plc, 500 – 85 Enterprise Blvd, Markham, ON L6G 0B5 Canada; 2 Montefiore Medical Center, Bronx, New York USA; 3 Allergan plc, Irvine, California USA; 4 IBM Watson Health Analytics, Bethesda, MD USA

**Keywords:** Hysterectomy, Leiomyoma, Management, Treatment pattern, Uterine fibroids

## Abstract

**Background:**

This study evaluated treatment patterns among women diagnosed with symptomatic uterine fibroids (UF) in the United States. Data were retrospectively extracted from the IBM Watson Health MarketScan® Commercial Claims and Encounters and Medicaid Multi-State databases.

**Methods:**

Women aged 18–64 years with ≥1 medical claim with a UF diagnosis (primary position, or secondary position plus ≥1 associated symptom) from January 2010 to June 2015 (Commercial) and January 2009 to December 2014 (Medicaid) were eligible; the first UF claim during these time periods was designated the index date. Data collected 12 months pre- and 12 and 60 months post-diagnosis included clinical/demographic characteristics, pharmacologic/surgical treatments, and surgical complications. Prevalence (2015) and cumulative incidence (Commercial, 2010–2015; Medicaid, 2009–2015) of symptomatic UF were estimated.

**Results:**

225,737 (Commercial) and 19,062 (Medicaid) women had a minimum of 12 months post-index continuous enrollment and were eligible for study. Symptomatic UF prevalence and cumulative incidence were: 0.57, 1.23% (Commercial) and 0.46, 0.64% (Medicaid). Initial treatments within 12 months post-diagnosis were surgical (Commercial, 36.7%; Medicaid, 28.7%), pharmacologic (31.7%; 53.0%), or none (31.6%; 18.3%). Pharmacologic treatments were most commonly non-steroidal anti-inflammatory drugs and oral contraceptives; hysterectomy was the most common surgical treatment. Of procedures of abdominal hysterectomy, abdominal myomectomy, uterine artery embolization, and ablation in the first 12 months post-index, 14.9% (Commercial) and 24.9% (Medicaid) resulted in a treatment-associated complication. Abdominal hysterectomy had the highest complication rates (Commercial, 18.5%; Medicaid, 31.0%).

**Conclusions:**

Off-label use of pharmacologic therapies and hysterectomy for treatment of symptomatic UF suggests a need for indicated non-invasive treatments for symptomatic UF.

## Background

Uterine fibroids (UF) are the most common tumor in women, affecting up to 70–80% of women by age 50 years [1]. Risk factors include race, age, family history of UF, and time since last birth [2]. Up to half of women with UF demonstrate clinical symptoms, including abnormal uterine bleeding (AUB), pelvic pressure/pain, bulk symptoms, and/or reproductive dysfunction [3, 4], which may lead to reduced quality of life [5, 6].

A lack of consensus exists within clinical guidelines regarding the most appropriate standard of care [7–10]. Treatment decisions are often determined by symptom severity, fibroid location, and patient characteristics [11].

Surgical treatments for UF include hysterectomy and uterine-sparing procedures: myomectomy, endometrial ablation, and uterine artery embolization (UAE) [4]. However, hysterectomy results in the loss of fertility and uterine-sparing procedures have increased risk for subsequent surgery [4].

No pharmacologic interventions are approved by the US Food and Drug Administration (FDA) for treatment of UF beyond a pre-operative indication. The gonadotropin-releasing hormone (GnRH) agonist leuprolide acetate is indicated only for pre-operative management of UF [12], while oral contraceptives, tranexamic acid, levonorgestrel intrauterine devices (IUDs), and non-steroidal anti-inflammatory drugs (NSAIDs) are used to treat symptoms, but are not indicated for UF treatment [4, 7–10, 13, 14]. As pharmacologic therapies provide only symptom relief, many patients undergo subsequent surgical treatments [10, 15].

Data regarding real-world treatment of women with symptomatic UF are limited [16]. To better understand contemporary treatment patterns, we evaluated patient clinical and demographic characteristics, epidemiologic characteristics of the disease, and treatment patterns in pre-menopausal women with symptomatic UF in the United States over a 5-year period.

## Methods

### Study design and data sources

This retrospective cohort study used administrative claims data from ~ 137.6 million people from the IBM Watson Health MarketScan® Commercial Claims and Encounters database (1995–2016) and ~ 44.2 million people from the MarketScan® Medicaid Multi-State database (1999–2015). The MarketScan® Commercial Claims and Encounters database contains the inpatient, outpatient, and outpatient prescription drug experience of US employees and their dependents, who are covered under a variety of fee-for-service and managed healthcare plans, including exclusive provider organizations, preferred provider organizations (PPOs), point of service plans, indemnity plans, and health maintenance organizations (HMOs). In contrast, Medicaid is a US healthcare program that assists low-income individuals in paying for their healthcare. The Medicaid Multi-State database contains the pooled healthcare experience of Medicaid enrollees, including records of inpatient services, inpatient admission, outpatient services, and prescription drug claims, as well as information on long-term care and other medical care. Data come from multiple states that are geographically dispersed. Both databases met US confidentiality requirements, including the Health Insurance Portability and Accountability Act of 1996, and were evaluated and certified by an independent third party, which performed quality control procedures to verify the information collected and provided linking of the databases [17]. Because the study used only de-identified patient records and did not involve the collection, use, or transmittal of individually identifiable data, Institutional Review Board approval was not necessary.

### Study population

**Fig. 1:**
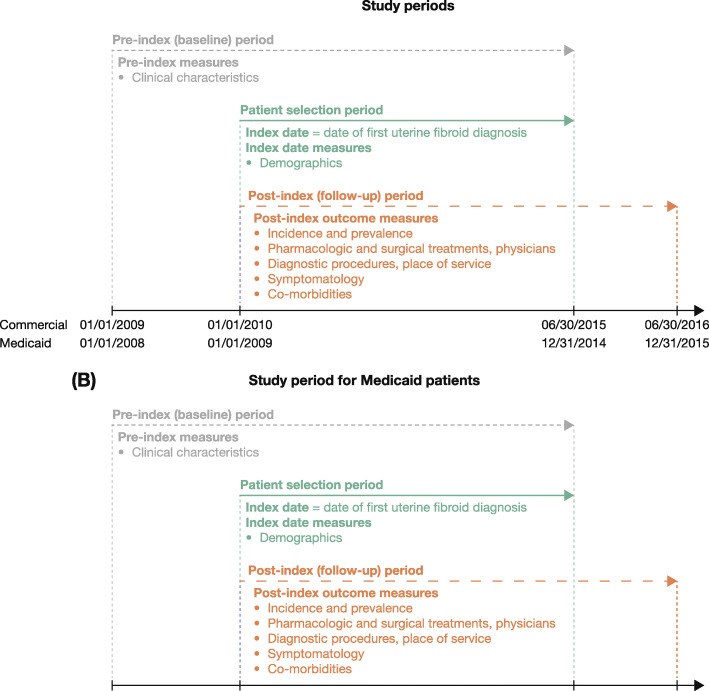
Study periods for the Commercial and Medicaid database cohorts

The study population included female patients aged 18–64 years who were diagnosed with symptomatic UF between January 1, 2010 and June 30, 2015 (Commercial database) and between January 1, 2009 and December 31, 2014 (Medicaid database). Symptomatic UF was defined as at least one inpatient or outpatient claim with a diagnosis code for UF in the primary position, or at least one inpatient or outpatient claim with a diagnosis code for UF in the secondary position and at least one diagnosis for an associated symptom (anemia due to blood loss, vaginal bleeding, or other menstrual bleeding disorders, pain associated with female genital organs, or urinary symptoms) in the primary position on the same date as the UF diagnosis claim. The index date was defined as the date of the first medical claim (inpatient or outpatient) with a diagnosis code for symptomatic UF. Data were included from the 12-month period before symptomatic UF diagnosis (pre-index period) and the 12- and 60-month periods after symptomatic UF diagnosis (post-index periods; inclusive of the index date) (Fig. 1).

Women were excluded from participation if they had a medical claim for menopause during the pre-index period or for malignant neoplasm during the entire study period. Patients with multiple claims on the same day for surgery (with the exception of multiple myomectomies, hysterectomies, and myomectomies and ablations), pharmacy, or pharmacy and surgery (with the exception of surgery and NSAID claims) were also excluded.

### Outcomes

#### Patient characteristics

Demographic characteristics collected on the index date included age, geographic region (Commercial patients only), race/ethnicity: White, Black, Hispanic, Other (Medicaid patients only), and health plan type.

#### Symptomatology and co-morbidities

Symptomatology and co-morbidities, defined by International Classification of Diseases (ICD)-9 and ICD-10 diagnosis codes (see Table S1, Additional file 1), were evaluated for the 12-month post-index period. Symptoms included anemia, vaginal bleeding/menstrual bleeding disorder, pain, urinary symptoms, heavy menstrual bleeding (HMB), and bulk symptoms (urinary frequency/incontinence, constipation, pelvic pressure/pain, abdominal distention, backache, leg pain, and increased abdominal girth). Fibroid-related co-morbidities included infertility, urinary tract infection, and constipation. Co-morbid endometriosis was assessed, and co-morbid depression and obesity were identified using the Elixhauser Comorbidity Index category [18]. The Elixhauser and Deyo-Charlson co-morbidity summary scores [19] were also recorded.

#### Epidemiologic characteristics of the disease

Diagnosed prevalence rates of symptomatic UF were calculated separately for each year (2010–2015 [Commercial database] and 2009–2015 [Medicaid database]). Prevalence was calculated as: (total cases of symptomatic UF reported in a given year) / (total included women aged 18–64 in the reported year). For the prevalence calculation, there was no requirement for any period of continuous enrollment before the date of the symptomatic UF claim; ≥1 day of enrollment on and after the claim was sufficient for inclusion.

Cumulative incidence rates of symptomatic UF were calculated for the combined period 2010–2015 (Commercial database) and 2009–2015 (Medicaid database). Incidence was calculated as: (total new cases of symptomatic UF in the reported years) / (total included women aged 18–64 in the reported years). Incident cases were defined as women with ≥12 months of continuous enrollment and no evidence of UF diagnosis in the 12 months before the symptomatic UF diagnosis date.

#### Treatment sequencing, timing, and utilization

Treatment sequencing was reported as the types of surgical or pharmacologic treatments received as initial or second treatments. Surgical/interventional treatments (defined using Current Procedural Terminology, ICD-9, and ICD-10 surgical procedure codes) included hysterectomy (recorded once according to the hierarchy: abdominal > laparoscopic > vaginal), myomectomy (recorded once according to the hierarchy: abdominal > laparoscopic > hysteroscopic > vaginal), UAE, ablation (including radiofrequency and magnetic resonance high-intensity focused ultrasound), and myomectomy and ablation. Pharmacologic treatments included NSAIDs (injectable ketorolac or oral), iron supplements (injectable or oral), GnRH agonists (leuprolide or others), hormonal contraceptives (including hormonal IUDs/levonorgestrel implants, oral contraceptives, and other contraceptives), aromatase inhibitors, tranexamic acid, selective estrogen receptor modulators, danazol, and dopamine promoters. Patients treated with NSAIDs proximal to surgical treatments were considered as receiving surgical treatment only. Treatment timings were recorded for the 12-month post-index period, and number of days from index date to first and second therapies was recorded. Results were presented by age, pre-index contraceptive use, and symptomatology. Data were collected on the use and timing of treatment with injectable NSAIDs within 7 days before or after UF-related surgical treatments or GnRH agonists at any time before or after UF-related surgical treatments.

The pattern of surgical and pharmacologic treatments was calculated as the percentage of patients having received each treatment for the 12- and 60-month post-index periods. Results were presented by age, pre-index contraceptive use, and symptomatology.

The occurrence of complications (defined using ICD-9 and ICD-10 codes) associated with abdominal hysterectomy, abdominal myomectomy, UAE, and ablation was calculated in the 12-month post-index group as the percentage of patients with at least one complication within 6 weeks (42 days) following the procedure. Complications were recorded as associated with the above four categories of surgery, and not further divided by, for example, type of myomectomy.

### Statistical analyses

Descriptive analyses were performed on all outcomes for both the Commercial and Medicaid databases. Frequencies and percentages were calculated for categorical variables, and means and standard deviations (SDs) were calculated for continuous variables.

## Results

### Patient characteristics

A total of 225,737 and 19,062 women with 12 months post-index eligibility met the inclusion criteria for symptomatic UF in the Commercial and Medicaid databases, respectively (Table 1). For the 12-month follow-up group, the mean (SD) age was 43.0 (7.1) years for the Commercial population and 38.9 (7.8) years for the Medicaid population. In the Commercial population, 44.6% of women resided in the South geographic region. In the Medicaid population, black women comprised 59.7% of the study population. The most common insurance plan types were HMOs (57.1%) in the Commercial population and PPOs (57.9%) in the Medicaid population. Demographics for the 60-month follow-up cohort were broadly similar to those reported for the 12-month follow-up cohort in both populations (data available upon request).

**Table 1:** Demographics, symptoms, and co-morbidities of the Commercial and Medicaid populations for the 12-month follow-up group

Characteristic	Commercial population	Medicaid population
	(*n* = 225,737)	(*n* = 19,062)
Mean (SD) age at index date, years	43.0 (7.1)	38.9 (7.8)
Age distributions, *n* ()		
18–24 years	1532 (0.7)	593 (3.1)
25–29 years	5485 (2.4)	1631 (8.6)
30–34 years	19,562 (8.7)	3385 (17.8)
35–29 years	40,403 (17.9)	4583 (24.0)
40–44 years	62,861 (27.8)	4340 (22.8)
45–49 years	58,717 (26.0)	2976 (15.6)
50–54 years	25,741 (11.4)	1017 (5.3)
55–59 years	7494 (3.3)	379 (2.0)
60–64 years	3942 (1.7)	158 (0.8)
Race, *n* ()		
Black	–	11,372 (59.7)
Hispanic	–	379 (2.0)
White	–	5438 (28.5)
Other	–	121 (0.6)
Unknown	–	1752 (9.2)
Insurance plan type, *n* (%)		
HMO	128,847 (57.1)	0 (0.0)
PPO	36,550 (16.2)	11,037 (57.9)
Other	51,856 (23.0)	8022 (42.1)
Unknown	8484 (3.8)	3 (< 0.1)
Geographic region, *n* (%)		
Northeast	45,430 (20.1)	–
North central	40,119 (17.8)	–
South	100,751 (44.6)	–
West	37,450 (16.6)	–
Unknown	1987 (0.9)	–
Symptoms^a^, *n* (%)		
Anemia	11,689 (5.2)	1609 (8.4)
Bulk symptoms	101,068 (44.8)	13,826 (72.5)
Abdominal distension	2849 (1.3)	325 (1.7)
Backache	30,717 (13.6)	6077 (31.9)
Constipation	7709 (3.4)	1958 (10.3)
Increased abdominal girth	5833 (2.6)	626 (3.3)
Leg pain	17,551 (7.8)	3523 (18.5)
Pelvic pressure/pain	60,825 (26.9)	9969 (52.3)
Urinary frequency/incontinence	17,695 (7.8)	3095 (16.2)
Vaginal bleeding, menstrual disorder	51,388 (22.8)	6987 (36.7)
HMB	96,879 (42.9)	9465 (49.7)
Urinary symptoms	19,009 (8.4)	3390 (17.8)
Pain	65,975 (29.2)	9782 (51.3)
Fibroid-related co-morbidities^a^, *n* (%)		
Infertility	7651 (3.4)	153 (0.8)
Urinary tract infection	18,348 (8.1)	3816 (20.0)
Constipation	7709 (3.4)	1958 (10.3)
Other co-morbidities^a^, *n* (%)		
Endometriosis	27,211 (12.1)	2520 (13.2)
Depression^b^	24,761 (11.0)	6012 (31.5)
Obesity^b^	23,119 (10.2)	5196 (27.3)
Mean (SD) Elixhauser score^a^	1.9 (3.4)	5.7 (6.3)
Mean (SD) Deyo-Charlson Index summary score^a^	0.2 (0.6)	0.6 (1.1)

*HMB* heavy menstrual bleeding, *HMO* health maintenance organization, *PPO* preferred provider organization, *SD* standard deviation

^a^12-month post-index period

^b^Determined using Elixhauser category

### Symptomatology and co-morbidities

In the 12 months post-index, bulk symptoms were the most common symptom (Commercial, 44.8%; Medicaid, 72.5%), with pelvic pressure/pain most frequently reported, while the most common fibroid-related co-morbidity was urinary tract infection (Commercial, 8.1%; Medicaid, 20.0%) (Table 1).

Women with symptomatic UF had mean (SD) Elixhauser co-morbidity summary scores of 1.9 (3.4) (Commercial) and 5.7 (6.3) (Medicaid) in the 12-month post-index period, and mean (SD) Deyo-Charlson Index summary scores of 0.2 (0.6) and 0.6 (1.1), respectively (Table 1).

### Epidemiologic characteristics of the disease

Between 2010 and 2015, the diagnosed prevalence of symptomatic UF ranged from 0.57–0.89% for the Commercial population. Between 2009 and 2015, the diagnosed prevalence ranged from 0.44–0.51% for the Medicaid population. The Commercial population had a 6-year cumulative incidence of 1.23% (343,341 cases from 2010 to 2015) and the Medicaid population had a 7-year cumulative incidence of 0.64% (32,525 cases from 2009 to 2015).

### Treatment sequencing and timing

**Fig. 2:**
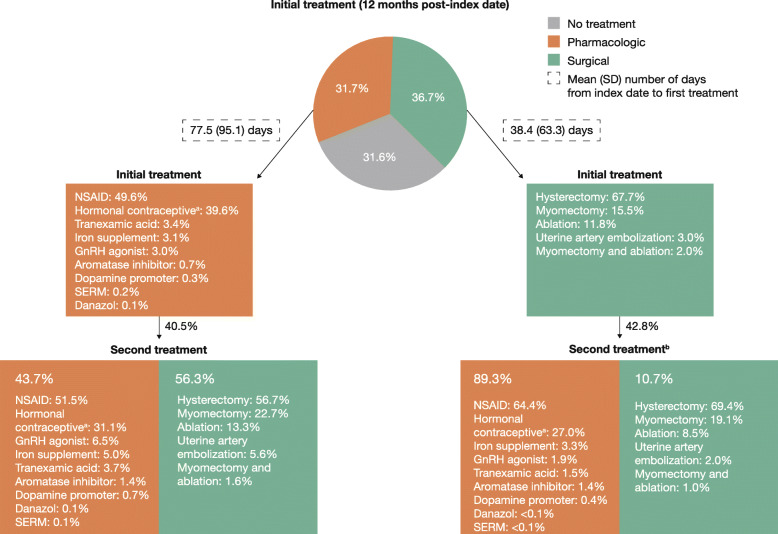
Treatments and time to treatment for the Commercial cohort; 12-month follow-up. ^a^Includes IUD/levonorgestrel implants, oral contraceptives, and other contraceptives; hormonal contraceptives were not mutually exclusive and a patient could receive > 1 type. ^b^Does not include women with hysterectomy as first-line procedure. GnRH: gonadotropin-releasing hormone; IUD: intrauterine device; NSAID: non-steroidal anti-inflammatory drug; SD: standard deviation; SERM: selective estrogen receptor modulator

**Fig. 3:**
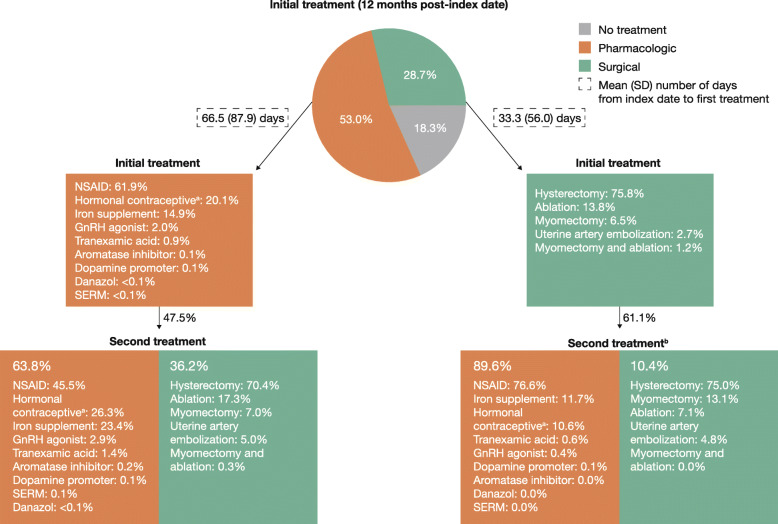
Treatments and time to treatment for the Medicaid cohort; 12-month follow-up. ^a^Includes IUD/levonorgestrel implants, oral contraceptives, and other contraceptives; hormonal contraceptives were not mutually exclusive and a patient could receive > 1 type. ^b^Does not include women with hysterectomy as first-line procedure. GnRH: gonadotropin-releasing hormone; IUD: intrauterine device; NSAID: non-steroidal anti-inflammatory drug; SD: standard deviation; SERM: selective estrogen receptor modulator

For the 12-month follow-up, initial treatments for Commercially insured women with symptomatic UF were divided relatively equally between surgical (36.7%), pharmacologic (31.7%), and no treatment (31.6%), whereas for Medicaid-insured women with symptomatic UF, the majority of women received pharmacologic treatment (53.0%), followed by surgical (28.7%) and no treatment (18.3%) (Figs. 2 and 3). Overall, the most common initial pharmacologic treatments were NSAIDs (15.7% [*n* = 35,489] and 32.8% [*n* = 6253] in the Commercial and Medicaid populations, respectively), followed by hormonal contraceptives (12.5% [*n* = 28,315] and 10.6% [*n* = 2029]). The most common initial surgical treatment was hysterectomy (Commercial, 24.8% [*n* = 56,072]; Medicaid, 21.8% [*n* = 4125]), followed by myomectomy (5.7% [*n* = 12,814]) in the Commercial population and ablation (4.0% [*n* = 756]) in the Medicaid population.

In the 12 months post-index, nearly half of women (Commercial, 41.1%; Medicaid, 49.1%) who received an initial pharmacologic or surgical treatment received a second treatment. Among Commercially insured women who received an initial pharmacologic treatment, 40.5% received a second treatment, most commonly surgery (56.3%), of whom 56.7% received a hysterectomy (Fig. 2). Among Commercially insured women who received surgery as initial treatment, 42.8% received a second treatment, most commonly pharmacologic treatment (89.3%) (Fig. 2). In the Medicaid population, a second treatment was received by 47.5% of those who received an initial pharmacologic treatment and 61.1% of those who received an initial surgical treatment; pharmacologic treatment was the most common second treatment in both groups (63.8 and 89.6%, respectively) (Fig. 3).

Injectable NSAID use, within 7 days before or after any surgical treatment, was low for symptomatic UF (Commercial, < 0.1%; Medicaid, 0.2%). GnRH agonist use was low at any time before (Commercial, 0.7%; Medicaid, 0.4%) or after (Commercial, 1.0%; Medicaid, 0.6%) any initial surgical treatment for symptomatic UF.

Among Commercially insured women with symptomatic UF, 25,771 women received oral contraceptives as initial treatment, of whom 5157 (20.0%) received a second treatment of surgery in the 12 months post-index. Of the 226 women who received an IUD as initial treatment, 24 women (10.6%) received surgery as a second treatment. Among Medicaid-insured women with symptomatic UF, 1613 women received oral contraceptives as the first treatment, of whom 196 (12.2%) received surgery as the second treatment, while 50 women received an IUD as initial treatment, of whom three (6.0%) received surgery as a second treatment.

Mean (SD) time from index date to first treatment was 56.5 (82.0) days (Commercial) and 54.8 (79.8) days (Medicaid), while time from index date to second treatment was 101.9 (100.5) days and 104.7 (99.6) days, respectively. Times to first surgical treatments were generally shorter than times to first pharmacologic treatments (Figs. 2 and 3).

### Treatment timing presented by age and pre-index contraceptive use

Among Commercially insured women with symptomatic UF presented by age, mean (SD) time to first treatment ranged from 53.1 (78.3) days (age 40–44 years) to 76.7 (96.9) days (age 55–59 years); time to first pharmacologic treatment generally increased with age, while time to first surgery remained largely consistent across the age groups (Table 2). For Medicaid-insured women with symptomatic UF, mean (SD) time to first treatment ranged from 51.5 (76.5) days (age 35–39 years) to 86.4 (98.6) days (age 60–64 years); time to first pharmacologic treatment and time to first surgery remained generally consistent across the age groups (Table 2). Time from index date to second treatment tended to decrease with age in both populations (Table 2).

**Table 2:** Any treatment received and time to treatment (Commercial and Medicaid populations; 12-month follow-up, by age)

	Age category								
	18–24 years	25–29 years	30–34 years	35–39 years	40–44 years	45–49 years	50–54 years	55–59 years	60–64 years
*Commercial population (n = 225,737)*									
*n*	1532	5485	19,562	40,403	62,861	58,717	25,741	7494	3942
Any treatment, *n* (%)	1114 (72.7)	4161 (75.9)	14,307 (73.1)	29,570 (73.2)	45,557 (72.5)	40,144 (68.4)	14,788 (57.4)	3220 (43.0)	1505 (38.2)
Mean (SD) time to first treatment^a^, days	57.0 (82.4)	63.6 (87.2)	62.5 (88.4)	57.3 (83.1)	53.1 (78.3)	53.8 (78.7)	58.7 (84.5)	76.7 (96.9)	76.4 (97.4)
Pharmacologic treatment	61.9 (85.5)	72.1 (92.2)	76.7 (96.5)	76.1 (94.7)	72.8 (91.4)	76.1 (93.7)	90.1 (101.1)	117.1 (107.6)	116.9 (107.4)
Surgical treatment	30.1 (55.8)	42.2 (68.6)	39.3 (67.1)	37.9 (63.5)	38.5 (63.2)	39.1 (62.9)	37.1 (62.2)	35.3 (61.5)	26.7 (49.9)
Mean (SD) time to second treatment, days	129.8 (102.2)	129.4 (108.4)	117.6 (105.2)	106.2 (102.3)	99.7 (99.2)	95.0 (97.2)	92.5 (97.9)	82.5 (94.6)	90.3 (98.7)
Pharmacologic^b^, *n* (%)									
Any	1052 (68.7)	3745 (68.3)	12,156 (62.1)	23,047 (57.0)	33,028 (52.5)	27,850 (47.4)	10,096 (39.2)	2362 (31.5)	1121 (28.4)
Aromatase inhibitor	5 (0.3)	98 (1.8)	311 (1.6)	391 (1.0)	226 (0.4)	29 (< 0.1)	4 (< 0.1)	2 (< 0.1)	0 (0.0)
Danazol	1 (0.1)	0 (0.0)	15 (0.1)	11 (< 0.1)	23 (< 0.1)	11 (< 0.1)	4 (< 0.1)	1 (< 0.1)	0 (0.0)
Dopamine promoter	2 (0.1)	20 (0.4)	117 (0.6)	146 (0.4)	118 (0.2)	76 (0.1)	24 (0.1)	6 (0.1)	0 (0.0)
GnRH agonist	45 (2.9)	185 (3.4)	617 (3.2)	929 (2.3)	1048 (1.7)	780 (1.3)	263 (1.0)	13 (0.2)	0 (0.0)
Hormonal contraceptive^c^	829 (54.1)	2351 (42.9)	6187 (31.6)	9206 (22.8)	10,290 (16.4)	6840 (11.6)	1583 (6.1)	70 (0.9)	8 (0.2)
Iron supplement	49 (3.2)	330 (6.0)	1002 (5.1)	1249 (3.1)	816 (1.3)	286 (0.5)	71 (0.3)	8 (0.1)	3 (0.1)
NSAID	493 (32.2)	2189 (39.9)	7714 (39.4)	16,310 (40.4)	25,289 (40.2)	22,482 (38.3)	8656 (33.6)	2243 (29.9)	1065 (27.0)
SERM	0 (0.0)	0 (0.0)	0 (0.0)	0 (0.0)	9 (< 0.1)	12 (< 0.1)	33 (0.1)	54 (0.7)	65 (1.6)
Tranexamic acid	13 (0.8)	66 (1.2)	261 (1.3)	610 (1.5)	1010 (1.6)	939 (1.6)	331 (1.3)	17 (0.2)	0 (0.0)
Surgical^b^, *n* (%)									
Any	254 (16.6)	1695 (30.9)	7399 (37.8)	18,524 (45.8)	32,251 (51.3)	29,177 (49.7)	10,175 (39.5)	1740 (23.2)	724 (18.4)
Ablation	15 (1.0)	114 (2.1)	650 (3.3)	2355 (5.8)	4932 (7.8)	4915 (8.4)	1477 (5.7)	94 (1.3)	17 (0.4)
Hysterectomy	23 (1.5)	366 (6.7)	2874 (14.7)	10,723 (26.5)	22,661 (36.0)	21,570 (36.7)	7822 (30.4)	1500 (20.0)	627 (15.9)
Myomectomy	219 (14.3)	1248 (22.8)	3908 (20.0)	5555 (13.7)	4703 (7.5)	2715 (4.6)	950 (3.7)	152 (2.0)	81 (2.1)
Myomectomy and ablation	5 (0.3)	30 (0.5)	120 (0.6)	371 (0.9)	722 (1.1)	767 (1.3)	217 (0.8)	20 (0.3)	4 (0.1)
Uterine artery embolization	4 (0.3)	18 (0.3)	177 (0.9)	577 (1.4)	1244 (2.0)	1243 (2.1)	326 (1.3)	37 (0.5)	4 (0.1)
*Medicaid population (n = 19,062)*									
*n*	593	1631	3385	4583	4340	2976	1017	379	158
Any treatment, *n* (%)	468 (78.9)	1380 (84.6)	2860 (84.5)	3828 (83.5)	3589 (82.7)	2389 (80.3)	728 (71.6)	234 (61.7)	98 (62.0)
Mean (SD) time to first treatment^a^, days	66.4 (87.1)	58.7 (86.8)	53.7 (80.4)	51.5 (76.5)	52.0 (76.2)	57.0 (80.9)	58.1 (82.8)	71.0 (82.1)	86.4 (98.6)
Pharmacologic treatment	70.9 (89.1)	70.0 (93.8)	65.2 (88.8)	64.1 (85.5)	64.6 (85.7)	66.1 (86.5)	69.7 (91.6)	82.1 (87.3)	109.4 (104.6)
Surgical treatment	23.5 (46.5)	23.9 (45.3)	29.8 (51.8)	31.6 (53.7)	33.1 (53.9)	42.5 (68.4)	37.0 (58.4)	35.7 (48.8)	31.6 (51.6)
Mean (SD) time to second treatment, days	143.0 (109.9)	119.5 (102.7)	111.1 (102.7)	99.0 (96.5)	98.1 (96.8)	101.9 (98.8)	97.8 (97.3)	99.0 (104.6)	106.9 (85.7)
Pharmacologic^b^, *n* (%)									
Any	459 (77.4)	1307 (80.1)	2622 (77.5)	3459 (75.5)	3196 (73.6)	2127 (71.5)	640 (62.9)	218 (57.5)	89 (56.3)
Aromatase inhibitor	1 (0.2)	1 (0.1)	3 (0.1)	4 (0.1)	5 (0.1)	0 (0.0)	0 (0.0)	0 (0.0)	0 (0.0)
Danazol	0 (0.0)	1 (0.1)	3 (0.1)	2 (< 0.1)	1 (< 0.1)	0 (0.0)	0 (0.0)	0 (0.0)	0 (0.0)
Dopamine promoter	0 (0.0)	1 (< 0.1)	3 (0.1)	2 (< 0.1)	8 (0.2)	1 (< 0.1)	0 (0.0)	1 (0.3)	0 (0.0)
GnRH agonist	18 (3.0)	41 (2.5)	71 (2.1)	88 (1.9)	74 (1.7)	54 (1.8)	8 (0.8)	2 (0.5)	0 (0.0)
Hormonal contraceptive^c^	258 (43.5)	573 (35.1)	819 (24.2)	826 (18.0)	532 (12.3)	252 (8.5)	28 (2.8)	4 (1.1)	0 (0.0)
Iron supplement	111 (18.7)	261 (16.0)	508 (15.0)	762 (16.6)	719 (16.6)	489 (16.4)	141 (13.9)	20 (5.3)	3 (1.9)
NSAID	349 (58.9)	1035 (63.5)	2191 (64.7)	2961 (64.6)	2756 (63.5)	1882 (63.2)	573 (56.3)	205 (54.1)	87 (55.1)
SERM	0 (0.0)	0 (0.0)	0 (0.0)	0 (0.0)	0 (0.0)	1 (< 0.1)	4 (0.4)	0 (0.0)	1 (0.6)
Tranexamic acid	1 (0.2)	11 (0.7)	37 (1.1)	40 (0.9)	46 (1.1)	21 (0.7)	2 (0.2)	0 (0.0)	0 (0.0)
Surgical^b^, *n* (%)									
Any	65 (11.0)	465 (28.5)	1308 (38.6)	2071 (45.2)	2060 (47.5)	1282 (43.1)	328 (32.3)	69 (18.2)	32 (20.3)
Ablation	15 (2.5)	70 (4.3)	212 (6.3)	344 (7.5)	366 (8.4)	194 (6.5)	30 (2.9)	3 (0.8)	0 (0.0)
Hysterectomy	28 (4.7)	323 (19.8)	973 (28.7)	1568 (34.2)	1575 (36.3)	1005 (33.8)	269 (26.5)	63 (16.6)	28 (17.7)
Myomectomy	25 (4.2)	81 (5.0)	142 (4.2)	163 (3.6)	124 (2.9)	57 (1.9)	18 (1.8)	4 (1.1)	4 (2.5)
Myomectomy and ablation	0 (0.0)	4 (0.2)	9 (0.3)	30 (0.7)	28 (0.6)	15 (0.5)	0 (0.0)	2 (0.5)	0 (0.0)
Uterine artery embolization	1 (0.2)	9 (0.6)	32 (0.9)	73 (1.6)	80 (1.8)	64 (2.2)	16 (1.6)	1 (0.3)	0 (0.0)

*GnRH* gonadotropin-releasing hormone, *IUD* intrauterine device, *NSAID* non-steroidal anti-inflammatory drug, *SD* standard deviation, *SERM* selective estrogen receptor modulator

^a^Time from index date

^b^Women could receive multiple treatments in the 12 months post-index; therefore, individual values may total > 100%

^c^Includes IUD/levonorgestrel implants, oral contraceptives, and other contraceptives; hormonal contraceptives were not mutually exclusive and a patient could receive > 1 type

Mean (SD) time to first treatment was shorter for women who received contraceptives in the 12 months pre-index (Commercial: 36.1 [59.9] days; Medicaid: 40.6 [66.8] days) compared to those who did not (Commercial: 62.8 [86.6] days; Medicaid: 57.9 [82.0] days); this pattern remained consistent for time to first pharmacologic treatment or surgical treatment (see Table S2, Additional file 2). Time from index date to second treatment was similar among those who did or did not receive contraceptives in the 12 months pre-index in both populations (see Table S2, Additional file 2).

Mean (SD) time to treatment was similar across UF-related symptoms, ranging from 48.6 (74.0) to 58.1 (81.8) days (Commercial) and 46.1 (69.1) to 54.1 (78.8) days (Medicaid) (see Table S3, Additional file 3).

### Treatment utilization presented by age, pre-index contraceptive use, and symptomatology

Proportions of women receiving any treatment in the 12 months post-index decreased with increasing age (Table 2). The proportion of women receiving any pharmacologic treatment decreased slightly with increasing age, while the proportion of women receiving any surgical treatment peaked at age 40–44 years. Uterine-sparing surgery (myomectomy, UAE, ablation, and myomectomy and ablation combined) peaked at age 25–29 years (Commercial, 25.7% [*n* = 1410]) and 40–44 years (Medicaid, 13.8% [*n* = 598]). In the Commercial population, the most common treatments were NSAIDs for all age groups (27.0–40.4%), except 18–24 years and 25–29 years, where hormonal contraceptives were the most common (54.1 and 42.9%, respectively). The second most common treatments were NSAIDs in women aged 18–24 years (32.2%) and 25–29 years (39.9%), hormonal contraceptives in women aged 30–34 years (31.6%), and hysterectomy for all age groups ≥35 years (15.9–36.7%). In the Medicaid population, the most common treatments among all age groups were NSAIDs (54.1–64.7%), followed by hormonal contraceptives for age groups 18–24 years and 25–29 years (43.5 and 35.1%, respectively), and hysterectomy for all age groups ≥30 years (16.6–36.3%).

Women with contraceptive use in the 12 months pre-index were more likely to undergo any treatment during the 12 months post-index compared to women with no pre-index contraceptive use (Commercial, 90.4% versus 63.6%; Medicaid, 91.1% versus 79.9%) (see Table S2, Additional file 2).

Treatment utilization according to symptomatology was similar to that observed in the overall population, with NSAIDs (Commercial, 43.8–49.6%; Medicaid, 66.5–70.6%) and hysterectomy (Commercial, 36.6–56.6%; Medicaid, 34.1–51.8%) the most common treatments received in the 12 months post-index. Women with anemia were more likely to undergo hysterectomy (Commercial, 56.6%; Medicaid, 51.8%) compared to women reporting other symptoms (Commercial, 36.6–45.7%; Medicaid, 34.1–43.2%) (see Table S3, Additional file 3).

### Treatment utilization over 60-month follow-up

Among women with longer continuous enrollment, the proportion of women receiving any treatment increased over the 60-month (5-year) follow-up, from 68.4% after 12 months to 86.3% (Commercial) and from 81.7 to 95.7% (Medicaid) (see Fig. S1, Additional file 4). The most common treatments by age group were similar for women with 12- and 60-month follow-up, with NSAIDs the most common treatment, followed by hysterectomy and hormonal contraceptives (data available upon request).

### Surgical treatment-associated complications

Of procedures of abdominal hysterectomy, abdominal myomectomy, UAE, and ablation in the first 12 months post-index, 14.9% (Commercial, *n* = 15,783/105,896) and 24.9% (Medicaid, *n* = 1982/7960) resulted in a treatment-associated complication. Abdominal hysterectomy was the most common surgical treatment for which complications were recorded (Commercial, 64.4% [*n* = 68,166]; Medicaid, 73.3% [*n* = 5832]), and was associated with the highest complication rates (18.5% [*n* = 12,603] and 31.0% [*n* = 1806], respectively). Post-operative adhesions (pelvic peritoneal; ICD-9614.6, ICD-10 N994) were the most commonly reported complication among those women who received an abdominal hysterectomy (Commercial, 9.5% [*n* = 6464]; Medicaid, 14.3% [*n* = 835]), followed by urinary tract infection (Commercial, 3.2%; Medicaid, 7.8%) and wound infection (Commercial, 1.6%; Medicaid, 4.6%). The overall mortality rate associated with abdominal hysterectomy was 0.36% (Commercial, 0.29%, *n* = 199; Medicaid, 1.17%, *n* = 68).

## Discussion

Symptomatic UF have a substantial impact on the health of women in the United States. In this study, annual diagnosed prevalence ranged from 0.57–0.89% and 0.44–0.51% for the Commercial (2010–2015) and Medicaid (2009–2015) databases, respectively. Women with symptomatic UF had low mean Deyo-Charlson Index summary scores, indicating they were generally healthy apart from their diagnosis with UF. Despite this, many women reported UF-related bulk symptoms and HMB, indicating a need for non-invasive treatments.

In this study, hysterectomy, NSAIDs, and hormonal contraceptives were the most frequently used treatments for symptomatic UF in both the Commercial and Medicaid populations. Similar to other studies [16, 20–22], hysterectomy was the most common surgery for UF and was often chosen over less invasive procedures. Hysterectomy is curative, but results in the loss of fertility and is associated with surgical risks, high costs, and concerns regarding loss of femininity [11, 16, 23, 24]. In this study, abdominal hysterectomy had the highest complication rates of those investigated in both populations. Less invasive surgeries are associated with recurrence of surgical treatments [4], which may indicate suboptimal effectiveness and may contribute to women choosing hysterectomy. In this study, mortality rates reported for the specific type of hysterectomy – abdominal hysterectomy – were 0.29% (Commercial) and 1.17% (Medicaid) during the 12-month post-index period. While the figure of 1.17% appears high, it is important to consider this in the context of the population under study. Medicaid provides healthcare for millions of the poorest and most vulnerable people in the US, including many with complex and costly needs; the elderly and people with disabilities account for one in four Medicaid enrollees [25]. Many studies have observed poorer outcomes and higher mortality rates in Medicaid enrollees compared to those with private healthcare insurance [26–28]. For example, an analysis of 89,460 patient-discharge records for inpatient shoulder arthroplasties from 2007 to 2014 found an increased probability of inpatient mortality (odds ratio [OR] 4.61; 95% confidence interval [CI] 2.18, 9.73; *P* < 0.001) and 30-day readmissions (OR 1.94; 95% CI 1.57, 2.38; *P* < 0.001) in Medicaid-insured patients compared to those with private, other, or Medicare insurance [29]. A large study of 295,572 patients who underwent total hip replacement found that Medicaid patients had a 125% increase in the odds of in-hospital mortality compared to those with private insurance (OR 2.25; 99% CI 1.01, 5.01) [27]. It is likely that other factors prevalent within the Medicaid population in the current investigation contributed to the raised mortality rate associated with abdominal hysterectomy, such as certain co-morbidities or disabilities.

Many women received pharmacologic therapies, even though none are FDA approved for symptomatic UF beyond a pre-operative indication. As in other reports [20, 21], NSAIDs were widely prescribed, likely reflecting their effectiveness in treating UF-associated pain. As previously reported [15, 20, 21], hormonal contraceptives were also commonly used. The accessibility, oral route of administration, and low cost may contribute to their use to treat UF-associated HMB or AUB despite the absence of indications or efficacy beyond short-term symptom control [30].

Considerations for treatment choice include age, patient preference, and desire for future fertility [31, 32], which were supported by these results. The sequence and timing of treatment for symptomatic UF varied between women of different age groups. Older women were less likely than younger women to receive treatment overall, perhaps reflecting “watchful waiting” as their chosen treatment option. Women in the youngest age groups were more likely to receive hormonal contraceptives and NSAIDs, presumably reflecting a desire to maintain fertility [31], whereas women ≥35 years (Commercial) or ≥ 30 years of age (Medicaid) were more likely to receive NSAIDs and hysterectomy as treatment. Uterine-sparing surgeries were highest in the 25–29-year age group (Commercial) and 40–44-year age group (Medicaid), suggesting that greater proportions of younger women are opting for uterine-sparing surgeries in the Commercial population compared to the Medicaid population.

Women who used contraceptives before diagnosis with symptomatic UF were more likely to undergo any treatment in the 12 months post-index, and had shorter times to first treatment, than women without a history of hormonal contraceptive use. This could reflect the use of contraceptives to treat generic bleeding symptoms without a formal diagnosis of UF, with progression of symptoms leading to diagnosis and subsequent treatment. The apparent failure of contraceptives as first treatment may suggest that they are not a sustainably effective treatment for UF symptoms. Of note, the levonorgestrel-releasing intrauterine system appeared to be a more effective first treatment than oral contraceptives, as evidenced by the lower percentage of women who required surgery as a second treatment after receiving initial therapy with a levonorgestrel-releasing intrauterine system versus oral contraceptives.

The limitations of this study include: identification of a study population based on administrative claims being subject to coding or entry errors (to minimize misclassification, women were required to have a UF diagnosis code in the primary position or at least one inpatient or outpatient claim with a UF diagnosis code in the secondary position plus at least one UF-associated symptom in the primary position on the same date as the UF diagnosis claim); reasons for observed treatment patterns cannot be established from claims data; generalizability of observations are limited by database type (i.e. the observed populations may not represent women with other forms of health insurance or without health insurance); data such as provider type were not available on all administrative claims. Also worthy of mention are differences between the two populations of women captured by the Medicaid and Commercial databases, in terms of demographics and symptomatology. For instance, most patients (57.1%) in the Commercial population had an HMO insurance plan, whereas most patients (57.9%) in the Medicaid population had a PPO insurance plan. These types of insurance plan differ in that HMO plans generally enable lower out-of-pocket expenses and require referrals to come from primary care physicians (PCPs), whereas PPO plans allow access to any in-network provider without needing PCP referral. Additionally, women in the Medicaid population had a high frequency of symptoms such as bulk symptoms (72.5%) and pain (51.3%), and of obesity (27.3%), compared with the Commercial population; such differences are likely due to known variations in ethnicity/race between the two populations. This study did not examine how myoma was diagnosed and whether treatment was related to the myoma; we aim to explore these factors in further research.

Despite these limitations, this study provides a detailed analysis of the management of women with a diagnosis of symptomatic UF in the United States between 2009 and 2016, including the large, robust MarketScan® Commercial Claims and Encounters database.

## Conclusions

The continued use of hysterectomy as the mainstay for treatment of symptomatic UF, along with the use of multiple non-surgical treatments to alleviate symptoms or the “watch and wait” approach, highlight the need for additional effective non-surgical treatment options.

## Supplementary information

**Additional file 1: Table S1**. ICD-9 and ICD-10 diagnosis codes for UF diagnosis, exclusion criteria, symptomatology, surgical/interventional procedures (including Current Procedural Terminology codes) and associated complications, and co-morbidities.

**Additional file 2: Table S2**. Any treatment received and time to treatment (12-month follow-up by pre-index hormonal contraceptive use).

**Additional file 3: Table S3**. Any treatment received for Commercial and Medicaid populations for the 12-month follow-up group by symptomatology.

**Additional file 4: Figure S1**. Proportion of women receiving any treatment over the 60-month follow-up for Commercial and Medicaid populations.

## Data Availability

The data sets supporting the conclusions of this article are available from Truven Health Analytics®, part of the IBM Watson Health™ business. Restrictions apply to the availability of these data, which were used under license for this study. Data are available from the authors with permission of Truven.© 2018 Truven Health Analytics, part of the IBM Watson Health business. All rights reserved.

## Abbreviations

AUB: Abnormal uterine bleeding
CI: Confidence interval
FDA: Food and Drug Administration
GnRH: Gonadotropin-releasing hormone
HMB: Heavy menstrual bleeding
HMO: Health maintenance organization
ICD: International Classification of Diseases
IUD: Intrauterine device
NSAID: Non-steroidal anti-inflammatory drug
OR: Odds ratio
PCP: Primary care physician
PPO: Preferred provider organization
SD: Standard deviation
UAE: Uterine artery embolization
UF: Uterine fibroids
