# Supplementary material for: Contemporary treatment utilization among women diagnosed with symptomatic uterine fibroids in the United States
Source: BMC Womens Health. 2020 Aug 13;20:174. doi: 10.1186/s12905-020-01005-6 (PMC7427077; doi:10.1186/s12905-020-01005-6)
Supplement: Supplementary file 1 — Additional file 1: Table S1. ICD-9 and ICD-10 diagnosis codes for UF diagnosis, exclusion criteria, symptomatology, surgical/interventional procedures (including Current Procedural Terminology codes) and associated complications, and co-morbidities. [file 12905_2020_1005_MOESM1_ESM.docx]

**Additional file 1**

**Table S1** ICD-9 and ICD-10 diagnosis codes for UF diagnosis, exclusion criteria, symptomatology, surgical/interventional procedures (including Current Procedural Terminology codes) and associated complications, and co-morbidities

|  | ICD-9-CM diagnosis code | ICD-10-CM diagnosis code | Current Procedural Terminology code |
| --- | --- | --- | --- |
| *Inclusion and exclusion diagnosis codes* | | | |
| Diagnosis of UF |  |  |  |
| Uterine leiomyoma | 218 | D25 |  |
| Submucous leiomyoma of uterus | 218.0 | D25.0 |  |
| Intramural leiomyoma of uterus | 218.1 | D25.1 |  |
| Subserous leiomyoma of uterus | 218.2 | D25.2 |  |
| Leiomyoma of uterus, unspecified | 218.9 | D25.9 |  |
| Exclusion criteria |  |  |  |
| Menopause | 627.XX, 256.31, V49.81 | N924, N950, N951, N952, N958, N959 |  |
| Malignant neoplasm | 140.XX–209.XX | C00–C14, C15–C26, C30–C39, C40–C41, C43–C44, C45–C49, C50–C58, C60–C63, C64–C68, C69–C72, C73–C75, C76–C80, C81–C96, C97 |  |
| UF-associated symptoms |  |  |  |
| Anemia due to blood loss | 280.0, 285.1 | D50.0, D62 |  |
| Vaginal bleeding, menstrual bleeding disorders | 623.8, 626.5, 626.6, 626.8, 626.9 | N89.8, N92.3, N92.4, N93.8, N93.9 |  |
| Pain associated with female genital organs | 625.0, 625.3, 625.5, 625.9 | N94.1, N94.4, N94.5, N94.6, N94.89, N94.9, R10.2 |  |
| Urinary symptoms | 596.5X, 788.2X, 788.3X (exclude 788.32 – male), 788.4X, 788.5, 788.6X, 788.9X | N32.81, N31.8, N31.2, N31.9, N36.44, N31.9, R33.9, R39.14, R33.8, R32, N39.41, N39.46, N39.42, N39.43, N39.44, N39.45, N39.490, N39.498, R35.0, R35.8, R35.1, R34, R39.13, R39.12, R39.15, R39.11, R39.16, R39.19, R39.81, R30.1, R39.2, R39.89 |  |
| Heavy menstrual bleeding | 626.2x, 627.0x | N92.0, N92.1, N92.5, N92.6 |  |
| Pregnancy | V22, v23, 650.xx, 651.xx | Z34.xx, Z3A.xx, Z33.1 |  |
| Bulk symptoms |  |  |  |
| Urinary frequency/incontinence | 788.1, 788.41, 788.20, 788.21, 788.29, 788.30, 788.30, 788.63, 788.3 | R350, R300, R309, R339, R3914, R330, R338, R32, R3915 |  |
| Constipation | 564.00, 564.01, 564.09 | K5900, K5901, K5909 |  |
| Pelvic pressure/pain | 625.0, 625.3, 789.00, 789.03, 789.04, 789.07, 789.09, 789.63, 789.64, 789.9 | N941, N944, N945, N946, R946, R10813, R10823, R10814, R10824, R198, R1030, R1031, R1032, R1084, R109 |  |
| Abdominal distention | 787.3 | R140, R141, R142, R143 |  |
| Backache | 724.1–724.5 | M5414–M5418, M5430, M5431, M5432, M5440, M5441, M5442, M545, M546, M5489, M549 |  |
| Leg pain | 729.5 | M79604, M79605, M79606, M79609, M79651, M79652, M79659, M79661, M79662, M79669, M79671, M79672, M79673, M79674, M79675, M79676 |  |
| Increased abdominal girth | 789.30–789.37, 789.39 | R1900–R1907, R1909 |  |
| UF-related co-morbidities |  |  |  |
| Infertility | 628.0–628.4, 628.8, 628.9 | N970, N971, N972, N978, N979, E230 |  |
| Endometriosis | 617.0–617.9 | N800–N809 |  |
| UTI | 599.0 | N390 |  |
| *Surgical procedure codes* | | | |
| Hysterectomy |  |  |  |
| Open/abdominal | 68.39, 68.49, 68.69 | 0UT90ZZ | 58150, 58152, 58180, 58200, 58210, 58240, 58953, 58954, 58956 |
| Vaginal, excluding laparoscopic | 68.59, 68.79 | 0UT97ZZ | 58260, 58262, 58263, 58267, 58270, 58275, 58280, 58285, 58290, 58291, 58292, 58293, 58294 |
| Laparoscopy | 68.31, 68.41, 68.51, 68.61, 68.71 | 0UT94ZZ, 0UT98ZZ, 0UT9FZZ | 58541, 58542, 58543, 58544, 58548, 58550, 58552, 58553, 58554, 58570, 58571, 58572, 58573 |
| Other | 68.9 |  |  |
| Myomectomy | 68.29 |  |  |
| Abdominal |  | 0U590ZZ, 0UB90ZZ | 58140, 58146 |
| Vaginal |  | 0U597ZZ, 0UB97ZZ | 58145 |
| Laparoscopy |  | 0U594ZZ, 0U598ZZ, 0UB94ZZ, 0UB98ZZ | 58545, 58546 |
| Hysteroscopy |  |  | 58561 |
| Uterine artery embolization | 38.8, 39.79, 99.29 (prior to 10/1/11; replaced with specific UAE codes 68.24, 68.25), 68.24, 68.25 | 04LE0DT, 04LE3DT, 04LE4DT, 04LF0DU, 04LF3DU, 04LF4DU, 04LE0CT, 04LE0ZT, 04LE3CT, 04LE3ZT, 04LE4CT, 04LE4ZT, 04LF0CU, 04LF0ZU, 04LF3CU, 04LF3ZU, 04LF4CU, 04LF4ZU | 37210 (code deleted in 2014; replaced with 37243), 37243 |
| Ablation | 68.23 | 0U5B0ZZ, 0U5B3ZZ, 0U5B4ZZ, 0U5B7ZZ, 0U5B8ZZ, 0UDB7ZZ, 0UDB8ZZ | 49203, 49204, 49205, 58353, 58356, 58563, 58674, 0071T, 0072T, 0404T, 0336T (prior 01/01/2017, replaced with 58674) |
| *Surgical/interventional complication codes* | | | |
| Abdominal hysterectomy |  |  |  |
| Hemorrhage with transfusion | 998.11 | N99820 |  |
| Hemorrhage without transfusion | 998.11 | N99820 |  |
| Infection – UTI | 590.10, 590.11, 590.3, 590.80, 590.81, 595.0, 595.3, 595.4, 595.81, 595.89, 595.59, 597.80, 597.81, 597.89, 599.0 | N3000, N3001, N3030, N3031, N3080, N3081, N3091, N390 |  |
| Infection – wound | 998.59 | T814XXA |  |
| Infection – vaginal infection | 616.10, 112.1, 131.01, 054.11 | N76.0, A54.02, A56.02, B37.3, A59.01, A60.04 |  |
| Infection – intraabdominal infection | 998.59 | T814XXA |  |
| Infection – sepsis/septicemia, bacteremia | 038.0, 038.10, 038.11, 038.12, 038.19, 038.2, 038.3, 038.40, 038.41, 038.42, 038.43, 038.44, 038.49, 038.8, 038.89, 790.7 | A400, A401, A403, A408, A409, A4101, A4102, A411, A412, A413, A414, A4150, A4151, A4152, A4153, A4159, A4181, A4189, A419 A5486, B377, R650, R6521, R7881 |  |
| Fever without known cause | 780.60, 780.62 | R50.9, R50.82 |  |
| Thromboembolic disease (DVT or PE) | 453.40, 453.41, 453.42, 453.8, 453.82, 451.11, 451.19, 451.81, 451.82, 451.83, 415.11, 415.12, 415.13, 415.19 | I82401, I82402, I82403, I82409, I82411, I82412, I82413, I82419, I82421, I82422, I82423, I82429, I82431, I82432, I82433, I82439, I82441, I82442, I82443, I82449, I82491, I82492, I82493, I82499 I824Y1, I824Y2, I824Y3 I824Y9, I824Z1, I824Z2, I824Z3, I824Z9, I8010, I8011, I8012, I8013, I80201, I80202, I80203, I80209, I80211, I80212, I80213, I80219, I80221, I80222, I80223, I80229, I80231, I80232, I80233, I80239, I80291, I80292, I80293, I80299, I82621, I82622, I82623, I82629 |  |
| Urinary tract issue – bladder injury | 867.0, 998.2 | S37.20XA, S37.21XA, S37.22XA, S37.23XA, S37.24XA, S37.25XA, S37.26XA, S37.27XA, S37.28XA, S37.29XA, N99.61 |  |
| Urinary tract issue – ureteral injury | 867.0, 998.2 | S37.10XA, S37.11XA, S37.12XA, S37.13XA, S37.14XA, S37.15XA, S37.16XA, S37.17XA, S37.18XA, S37.19XA, N99.61 |  |
| Gastrointestinal tract issue – ileus | 560.1, 997.49 | K56.7, K91.89 |  |
| Gastrointestinal tract issue – bowel injury | 863.20–863.59 | S36.400A–S36.599A, K91.72 |  |
| Gastrointestinal tract issue – bowel obstruction | 560.9, 997.49 | K91.3 |  |
| Vaginal cuff dehiscence | 998.32 | T81.31XXA |  |
| Post-operative adhesions (pain/pelvic or abdominal) | 614.6 | N994 |  |
| Earlier menopause | 256.31, 256.2 | E28.310, E28.311, E28.312, E28.313, E28.314, E28.315, E28.316, E28.317, E28.318, E28.319, E89.40 |  |
| Mortality | 799.9 | R99 |  |
| Hematoma | 998.12 | N99.820, N99.840 |  |
| Abdominal myomectomy |  |  |  |
| Hemorrhage with transfusion | 998.11 | N99820 |  |
| Hemorrhage without transfusion | 998.11 | N99820 |  |
| Infection – UTI | 590.10, 590.11, 590.3, 590.80, 590.81, 595.0, 595.3, 595.4, 595.81, 595.89, 595.59, 597.80, 597.81, 597.89, 599.0 | N3000, N3001, N3030, N3031, N3080, N3081, N3091, N390 |  |
| Infection – wound | 998.59 |  |  |
| Infection – vaginal infection | 616.10, 112.1, 131.01, 054.11 | N76.0, A54.02, A56.02, B37.3, A59.01, A60.04 |  |
| Infection – intraabdominal infection | 998.59 | T814XXA |  |
| Infection – sepsis/septicemia, bacteremia | 038.0, 038.10, 038.11, 038.12, 038.19, 038.2, 038.3, 038.40, 038.41, 038.42, 038.43, 038.44, 038.49, 038.8, 038.89, 790.7 | A400, A401, A403, A408, A409, A4101, A4102, A411, A412, A413, A414, A4150, A4151, A4152, A4153, A4159, A4181, A4189, A419, A5486, B377, R650, R6521, R7881 |  |
| Fever without known cause | 780.60, 780.62 | R50.9, R50.82 |  |
| Thromboembolic disease (DVT or PE) | 453.40, 453.41, 453.42, 453.8, 453.82, 451.11, 451.19, 451.81, 451.82, 451.83, 415.11, 415.12, 415.13, 415.19 | I82401, I82402, I82403, I82409, I82411, I82412, I82413, I82419, I82421, I82422, I82423, I82429, I82431, I82432, I82433, I82439, I82441, I82442, I82443, I82449, I82491, I82492, I82493, I82499, I824Y1, I824Y2, I824Y3, I824Y9, I824Z1, I824Z2, I824Z3, I824Z9, I8010, I8011, I8012, I8013, I80201, I80202, I80203, I80209, I80211, I80212, I80213, I80219, I80221, I80222, I80223, I80229, I80231, I80232, I80233, I80239, I80291, I80292, I80293, I80299, I82621, I82622, I82623, I82629 |  |
| Urinary tract issue – bladder injury | 867.0, 998.2 | S37.20XA, S37.21XA, S37.22XA, S37.23XA, S37.24XA, S37.25XA, S37.26XA, S37.27XA, S37.28XA, S37.29XA, N99.61 |  |
| Gastrointestinal tract issue – ileus | 560.1, 997.49 | K56.7, K91.89 |  |
| Gastrointestinal tract issue – bowel injury | 863.20–863.59 | S36.400A–S36.599A, K91.72 |  |
| Gastrointestinal tract issue – bowel obstruction | 560.9, 997.49 | K91.3 |  |
| Post-operative adhesions (pain/pelvic or abdominal) | 614.6 | N994 |  |
| Earlier menopause | 256.31, 256.2 | E28.310, E28.311, E28.312, E28.313, E28.314, E28.315, E28.316, E28.317, E28.318, E28.319, E89.40 |  |
| Mortality | 799.9 | R99 |  |
| Hysterectomy | 68.39, 68.49, 68.69, 68.59, 68.79, 68.31, 68.41, 68.51, 68.61, 68.71, 68.9 | 0UT90ZZ, 0UT97ZZ, 0UT94ZZ, 0UT98ZZ, 0UT9FZZ |  |
| Uterine artery embolization |  |  |  |
| Periprocedural – groin hematoma | 998.11 | L76.22, L76.32 |  |
| Periprocedural – arterial thrombosis | 444.22 | I74.3 |  |
| Periprocedural – infrequently pseudoaneurysm | 442.3, 442.89 | I72.4, I72.8 |  |
| “Postembolization syndrome” (fever, nausea, pain, malaise) | 780.62, 787.01, 338.18, 780.79 | R50.82, R11.0, G89.18, R53.81 |  |
| Vaginal discharge | 623.5 | N89.8 |  |
| Prolapsed fibroid | 618.89, 618.89, 618.1 | N81.89, N81.2, N81.3, N81.4 |  |
| Ablation |  |  |  |
| Surgical tears, perforations, or lacerations | 998.2 | N997.1 |  |
| Other complication affecting other specified body systems/Other intraoperative complications of genitourinary system | 997.99 | N99.81 |  |
| *Elixhauser Comorbidity Index codes* | | | |
| Congestive heart failure | 398.91, 402.01, 402.11. 402.91, 404.01, 404.03, 404.11, 404.13, 404.91, 404.93, 425.4–425.9, 428.x | I09.9, I11.0, I13.0, I13.2, I25.5, I42.0, 142.5–I42.9, I43.x, I50.x, P29.0 |  |
| Cardiac arrhythmias | 426.0, 426.13, 426.7, 426.9, 426.10, 426.12, 427.0–427.4, 427.6–427.9, 785.0, 996.01, 996.04, V45.0, V53.3 | I44.1–I44.3, I45.6, I45.9, I47.x–I49.x, ROO.O, ROO.1, ROO.8, T82.1, Z45.0, Z95.0 |  |
| Valvular disease | 093.2, 394.x–397.x, 424.x, 746.3–746.6, V42.2, V43.3 | A52.0, I05.x–I08.x, I09.1, I09.8, I34.x–I39.x, Q23.0– Q23.3, Z95.2, Z95.4 |  |
| Pulmonary circulation disorders | 415.0, 415.1, 416.x, 417.0, 417.8, 417.9 | I26.x, I27.x, I28.0, I28.8, I28.9 |  |
| Peripheral vascular disorders | 093.0, 437.3, 440.x, 441.x, 443.1–443.9, 447.1, 557.1 557.9, V43.4 | I70.x, I71.x, I73.1, I73.8, I73.9, I77.1, I79.0, I79.2, K55.1, K55.8, K55.9, Z95.8, Z95.9 |  |
| Hypertension, uncomplicated | 401.x | I10.x |  |
| Hypertension, complicated | 402.x–405.x | I11.x–I13.x, I15.x |  |
| Paralysis | 334.1, 342.x, 343.x, 344.0–344.6, 344.9 | G04.1, G11.4, G80.1, G80.2, G81.x, G82.x, G83.0–G83.4, G83.9 |  |
| Other neurological disorders | 331.9, 332.0, 332.1, 333.4, 333.5, 333.92, 334.x– 335.x, 336.2, 340.x, 341.x, 345.x, 348.1, 348.3, 780.3, 784.3 | G10.x–G13.x, G20.x–G22.x, G25.4, G25.5, G31.2, G31.8, G31.9, G32.x, G35.x–G37.x, G40.x, G41.x, G93.1, G93.4, R47.0, R56.x |  |
| Chronic pulmonary disease | 416.8, 416.9, 490.x–505.x, 506.4, 508.1, 508.8 | I27.8, 127.9, J40.x–J47.x, J60.x–J67.x, J68.4, J70.1, J70.3 |  |
| Diabetes, uncomplicated | 250.0–250.3 | E10.0, E10.1, E10.9, E11.0, E11.1, E11.9, E12.0, E12.1, E12.9, E13.0, E13.1, E13.9, E14.0, E14.1, E14.9 |  |
| Diabetes, complicated | 250.4–250.9 | E10.2–E10.8, E11.2–E11.8, E12.2–E12.8, E13.2–E13.8, E14.2–E14.8 |  |
| Hypothyroidism | 240.9, 243.x, 244.x, 246.1, 246.8 | E00.x–E03.x, E89.0 |  |
| Renal failure | 403.01, 403.11, 403.91, 404.02, 404.03, 404.12, 404.13, 404.92, 404.93, 585.x, 586.x, 588.0, V42.0, V45.1, V56.x | I12.0, I13.1, N18.x, NI9.x, N25.0, Z49.0–Z49.2, Z94.0, Z199.2 |  |
| Liver disease | 070.22, 070.23, 070.32, 070.33, 070.44, 070.54, 070.6, 070.9, 456.0–456.2, 570.x, 571.x, 572.2–572.8, 573.3, 573.4, 573.8, 573.9, V42.7 | B18.x, I85.x, I86.4, I98.2, K70.x, K71.1, K71.3–K71.5, K71.7, K72.x–K74.x, K76.0, K76.2–K76.9, Z94.4 |  |
| Peptic ulcer disease excluding bleeding | 531.7, 531.9, 532.7, 532.9, 533.7, 533.9, 534.7, 534.9 | K25.7, K25.9, K26.7, K26.9, K27.7, K27.9, K28.7, K28.9 |  |
| AIDS/HIV | 042.x–044.x | B20.x–B22.x, B24.x |  |
| Lymphoma | 200.x–202.x, 203.0, 238.6 | C81.x–C85.x, C88.x, C96.x, C90.0, C90.2 |  |
| Metastatic cancer | 196.x–199.x | C77.x–C80.x |  |
| Solid tumor without metastasis | 140.x–172.x, 174.x–195.x | C00.x–C26.x, C30.x, C34.x, C37.x–C41.x, C43.x, C45.x–C58.x, C60.x–C76.x, C97.x |  |
| Rheumatoid arthritis/collagen vascular diseases | 446.x, 701.0, 710.0–710.4, 710.8, 710.9, 711.2, 714.x, 719.3, 720.x, 725.x, 728.5, 728.89, 729.30 | L94.0, L94.1, L94.3, M05.x, M06.x, M08.x, M12.0, M12.3, M30.x, M31.0–M31.3, M32.x–M35.x, M45.x, M46.1, M46.8, M46.9 |  |
| Coagulopathy | 286.x, 287.1, 287.3–287.5 | D65–D68.x, D69.1, D69.3–D69.6 |  |
| Obesity | 278.0 | E66.x |  |
| Weight loss | 260.x–263.x, 783.2, 799.4 | E40.x–E46.x, R63.4, R64 |  |
| Fluid and electrolyte disorders | 253.6, 276.x | E22.2, E86.x, E87.x |  |
| Blood loss anemia | 280.0 | D50.0 |  |
| Deficiency anemia | 280.1–280.9, 281.x | D50.8, D50.9, D51.x–D53.x |  |
| Alcohol abuse | 265.2, 291.1–291.3, 291.5 291.9, 303.0, 303.9, 305.0, 357.5, 425.5, 535.3, 571.0–571.3, 980.x, V11.3 | F10, E52, G62.1, I42.6, K29.2, K70.0, K70.3, K70.9, T51.x, Z50.2, Z71.4, Z72.1 |  |
| Drug abuse | 292.x, 304.x, 305.2–305.9, V65.42 | F11.x–F16.x, F18.x, F19.x, Z71.5, Z72.2 |  |
| Psychoses | 293.8, 295.x, 296.04, 296.14, 296.44, 296.54, 297.x, 298.x | F20.x, F22.x–F25.x, F28.x, F29.x, F30.2, F31.2, F31.5 |  |
| Depression | 296.2, 296.3, 296.5, 300.4, 309.x, 311 | F20.4, F31.3–F31.5, F32.x, F33.x, F34.1, F41.2, F43.2 |  |

AIDS: acquired immunodeficiency syndrome; DVT: deep vein thrombosis; HIV: human immunodeficiency virus; ICD‑9‑CM: International Classification of Diseases, Ninth Revision, Clinical Modification; ICD-10-CM: International Classification of Diseases, Tenth Revision, Clinical Modification; PE: pulmonary embolism; UAE: uterine artery embolization; UF: uterine fibroids; UTI: urinary tract infection
