# Supplementary material for: Contemporary treatment utilization among women diagnosed with symptomatic uterine fibroids in the United States
Source: BMC Womens Health. 2020 Aug 13;20:174. doi: 10.1186/s12905-020-01005-6 (PMC7427077; doi:10.1186/s12905-020-01005-6)
Supplement: Supplementary file 2 — Additional file 2: Table S2. Any treatment received and time to treatment (12-month follow-up by pre-index hormonal contraceptive use). [file 12905_2020_1005_MOESM2_ESM.docx]

**Additional file 2**

**Table S2** Any treatment received and time to treatment (12-month follow-up by pre-index hormonal contraceptive use)

|  | Commercial population (*n* = 225,737) | | Medicaid population (*n* = 19,062) | |
| --- | --- | --- | --- | --- |
|  | No pre-index hormonal contraceptive use (*n* = 185,643) | Pre-index hormonal contraceptive use (*n* = 40,094) | No pre-index hormonal contraceptive use (*n* = 16,002) | Pre-index hormonal contraceptive use (*n* = 3060) |
| Any treatment, *n* (%) | 118,135 (63.6) | 36,231 (90.4) | 12,786 (79.9) | 2788 (91.1) |
| Mean (SD) time to first treatment^a^, days  Pharmacologic treatment  Surgical treatment | 62.8 (86.6) 94.7 (102.9) 41.1 (65.3) | 36.1 (59.9) 43.2 (64.6) 22.5 (46.8) | 57.9 (82.0) 71.3 (90.8) 35.1 (57.6) | 40.6 (66.8) 47.6 (72.4) 21.3 (42.7) |
| Mean (SD) time to second treatment, days | 103.1 (101.7) | 98.7 (97.4) | 104.9 (99.6) | 103.7 (99.5) |
| Pharmacologic^b^, *n* (%)  Any  Aromatase inhibitor  Danazol  Dopamine promoter  GnRH agonist  Hormonal contraceptive^c^  Iron supplement  NSAID  SERM  Tranexamic acid | 82,941 (44.7) 843 (0.5) 56 (<0.1) 387 (0.2) 2817 (1.5) 14,058 (7.6) 2889 (1.6) 70,174 (37.8) 164 (0.1) 2724 (1.5) | 31,516 (78.6) 223 (0.6) 10 (<0.1) 122 (0.3) 1063 (2.7) 23,297 (58.1) 925 (2.3) 16,267 (40.6) 9 (<0.1) 523 (1.3) | 11,499 (71.9) 11 (0.1) 6 (<0.1) 12 (0.1) 277 (1.7) 1712 (10.7) 2466 (15.4) 10,066 (62.9) 5 (<0.1) 135 (0.8) | 2618 (85.6) 3 (0.1) 1 (<0.1) 4 (0.1) 79 (2.6) 1580 (51.6) 548 (17.9) 1973 (64.5) 1 (<0.1) 23 (0.8) |
| Surgical^b^, *n* (%)  Any  Ablation   Hysterectomy  Myomectomy  Myomectomy and ablation  Uterine artery embolization | 82,969 (44.7) 11,926 (6.4) 57,259 (30.8) 13,877 (7.5) 1672 (0.9)  2919 (1.6) | 18,970 (47.3) 2643 (6.6) 10,907 (27.2) 5654 (14.1) 584 (1.5) 711 (1.8) | 6526 (40.8) 1000 (6.2) 5040 (31.5) 472 (2.9) 71 (0.4) 241 (1.5) | 1154 (37.7) 234 (7.6) 792 (25.9) 146 (4.8) 17 (0.6) 35 (1.1) |

^a^Time from index date
^b^Women could receive multiple treatments in the 12 months post-index; therefore, individual values may total >100%
^c^Includes IUD/levonorgestrel implants, oral contraceptives, and other contraceptives; hormonal contraceptives were not mutually exclusive and a patient could receive >1 type
GnRH: gonadotropin-releasing hormone; IUD: intrauterine device; NSAID: non-steroidal anti-inflammatory drug; SD: standard deviation; SERM: selective estrogen receptor modulator
