# Supplementary material for: Contemporary treatment utilization among women diagnosed with symptomatic uterine fibroids in the United States
Source: BMC Womens Health. 2020 Aug 13;20:174. doi: 10.1186/s12905-020-01005-6 (PMC7427077; doi:10.1186/s12905-020-01005-6)
Supplement: Supplementary file 3 — Additional file 3: Table S3. Any treatment received for Commercial and Medicaid populations for the 12-month follow-up group by symptomatology. [file 12905_2020_1005_MOESM3_ESM.docx]

**Additional file 3**

**Table S3** Any treatment received for Commercial and Medicaid populations for the 12-month follow-up group by symptomatology

|  | UF-related symptom | | | | | |
| --- | --- | --- | --- | --- | --- | --- |
|  | Anemia | Vaginal bleeding | Pain | Urinary symptoms | HMB | Bulk symptoms |
| *Commercial population (n = 225,737)* | | | | | | |
| *n* | 11,689 | 51,388 | 65,975 | 19,009 | 96,879 | 101,068 |
| Any treatment, *n* (%) | 10,332 (88.4) | 40,640 (79.1) | 52,721 (79.9) | 14,568 (76.6) | 81,074 (83.7) | 76,299 (75.5) |
| Mean (SD) time to first treatment^a^, days  Pharmacologic treatment  Surgical treatment | 53.3 (75.7) 58.5 (81.2) 50.5 (72.5) | 54.7 (79.3) 69.3 (90.0) 43.9 (68.5) | 53.2 (78.4) 70.4 (90.6) 40.9 (65.8) | 58.1 (81.8) 78.4 (95.0) 43.6 (67.2) | 48.6 (74.0) 64.6 (86.4) 40.0 (64.7) | 58.0 (83.0) 79.5 (96.2) 39.8 (64.6) |
| Pharmacologic^b^, *n* (%)  Any  Aromatase inhibitor  Danazol  Dopamine promoter  GnRH agonist  Hormonal contraceptive^c^  Iron supplement  NSAID  SERM  Tranexamic acid | 7032 (60.2) 18 (0.2) 7 (0.1) 17 (0.1) 428 (3.7) 1870 (15.9) 200 (1.7) 5797 (49.6) 3 (<0.1) 414 (3.5) | 29,098 (56.6) 152 (0.3) 18 (<0.1) 92 (0.2) 947 (1.8) 9858 (19.2) 790 (1.5) 22,522 (43.8) 11 (<0.1) 1171 (2.3) | 38,757 (58.7) 344 (0.5) 22 (<0.1) 113 (0.2) 1250 (1.9) 11,041 (16.7) 1126 (1.7) 31,899 (48.4) 40 (0.1) 877 (1.3) | 10,778 (56.7) 57 (0.3) 2 (<0.1) 38 (0.2) 303 (1.6) 2811 (14.8) 246 (1.3) 8939 (47.0) 18 (0.1) 255 (1.3) | 55,312 (57.1) 222 (0.2) 36 (<0.1) 134 (0.1) 1795 (1.9) 15,376 (15.9) 988 (1.0) 44,727 (46.2) 23 (<0.1) 2550 (2.6) | 57,801 (57.2) 462 (0.5) 33 (<0.1) 221 (0.2) 1779 (1.8) 16,286 (16.1) 1677 (1.7) 47,361 (46.9) 85 (0.1) 1404 (1.4) |
| Surgical^b^, *n* (%)  Any  Ablation  Hysterectomy  Myomectomy  Myomectomy and ablation  Uterine artery embolization | 8970 (76.7) 984 (8.4) 6617 (56.6) 1563 (13.4) 172 (1.5) 286 (2.4) | 30,086 (58.5) 6355 (12.4) 19,664 (38.3) 5057 (9.8) 845 (1.6) 816 (1.6) | 38,711 (58.7) 3724 (5.6) 29,235 (44.3) 6096 (9.2) 564 (0.9) 1024 (1.6) | 10,601 (55.8) 977 (5.1) 8209 (43.2) 1369 (7.2) 125 (0.7) 419 (2.2) | 66,027 (68.2) 13,066 (13.5) 44,300 (45.7) 10,104 (10.4) 1926 (2.0) 1967 (2.0) | 51,515 (51.0) 6271 (6.2) 37,020 (36.6) 8496 (8.4) 923 (0.9) 1647 (1.6) |
| *Medicaid population (n = 19,062)* | | | | | | |
| *n* | 1609 | 6987 | 9782 | 3390 | 9465 | 13,826 |
| Any treatment, *n* (%) | 1475 (91.7) | 6117 (87.5) | 8514 (87.0) | 2948 (87.0) | 8479 (89.6) | 11,868 (85.8) |
| Mean (SD) time to first treatment^a^, days  Pharmacologic treatment  Surgical treatment | 46.1 (69.1) 49.3 (73.5) 41.7 (62.3) | 51.7 (75.3) 58.7 (82.1) 38.6 (58.6) | 51.0 (75.9) 60.6 (83.8) 35.3 (57.3) | 53.3 (79.4) 64.8 (87.0) 33.0 (58.3) | 47.2 (71.8) 57.3 (81.2) 35.0 (56.2) | 54.1 (78.8) 65.6 (86.7) 33.3 (56.1) |
| Pharmacologic^b^, *n* (%)  Any  Aromatase inhibitor  Danazol  Dopamine promoter  GnRH agonist  Hormonal contraceptive^c^  Iron supplement  NSAID  SERM  Tranexamic acid | 1312 (81.5) 0 (0.0) 0 (0.0) 1 (0.1) 47 (2.9) 246 (15.3) 568 (35.3) 1070 (66.5) 0 (0.0) 15 (0.9) | 5559 (79.6) 7 (0.1) 4 (0.1) 8 (0.1) 189 (2.7) 1532 (21.9) 1379 (19.7) 4767 (68.2) 0 (0.0) 81 (1.2) | 7711 (78.8) 8 (0.1) 6 (0.1) 8 (0.1) 224 (2.3) 1763 (18.0) 1466 (15.0) 6823 (69.8) 1 (<0.1) 87 (0.9) | 2696 (79.5) 3 (0.1) 2 (0.1) 2 (0.1) 58 (1.7) 620 (18.3) 548 (16.2) 2392 (70.6) 1 (<0.1) 32 (0.9) | 7464 (78.9) 6 (0.1) 5 (0.1) 6 (0.1) 199 (2.1) 1656 (17.5) 1744 (18.4) 6458 (68.2) 2 (<0.1) 128 (1.4) | 10,836 (78.4) 10 (0.1) 6 (<0.1) 11 (0.1) 273 (2.0) 2390 (17.3) 2217 (16.0) 9564 (69.2) 4 (<0.1) 120 (0.9) |
| Surgical^b^, *n* (%)  Any  Ablation  Hysterectomy  Myomectomy  Myomectomy and ablation  Uterine artery embolization | 1023 (63.6) 121 (7.5) 833 (51.8) 84 (5.2) 13 (0.8) 47 (2.9) | 3326 (47.6) 726 (10.4) 2383 (34.1) 283 (4.1) 54 (0.8) 127 (1.8) | 4681 (47.9) 597 (6.1) 3808 (38.9) 319 (3.3) 34 (0.3) 130 (1.3) | 1568 (46.3) 211 (6.2) 1259 (37.1) 102 (3.0) 19 (0.6) 53 (1.6) | 5558 (58.7) 1140 (12.0) 4092 (43.2) 403 (4.3) 80 (0.8) 181 (1.9) | 6023 (43.6) 920 (6.7) 4718 (34.1) 411 (3.0) 67 (0.5) 206 (1.5) |

^a^Time from index date
^b^Women could receive multiple treatments in the 12 months post-index; therefore, individual values may total >100%
^c^Includes IUD/levonorgestrel implants, oral contraceptives, and other contraceptives; hormonal contraceptives were not mutually exclusive and a patient could receive >1 type
GnRH: gonadotropin-releasing hormone; HMB: heavy menstrual bleeding; IUD: intrauterine device; NSAID: non-steroidal anti-inflammatory drug; SD: standard deviation; SERM: selective estrogen receptor modulator; UF: uterine fibroids
