# Supplementary material for: Contemporary treatment utilization among women diagnosed with symptomatic uterine fibroids in the United States
Source: BMC Womens Health. 2020 Aug 13;20:174. doi: 10.1186/s12905-020-01005-6 (PMC7427077; doi:10.1186/s12905-020-01005-6)

**Additional file 4**

**Fig. S1** Proportion of women receiving any treatment over the 60-month follow-up for Commercial and Medicaid populations


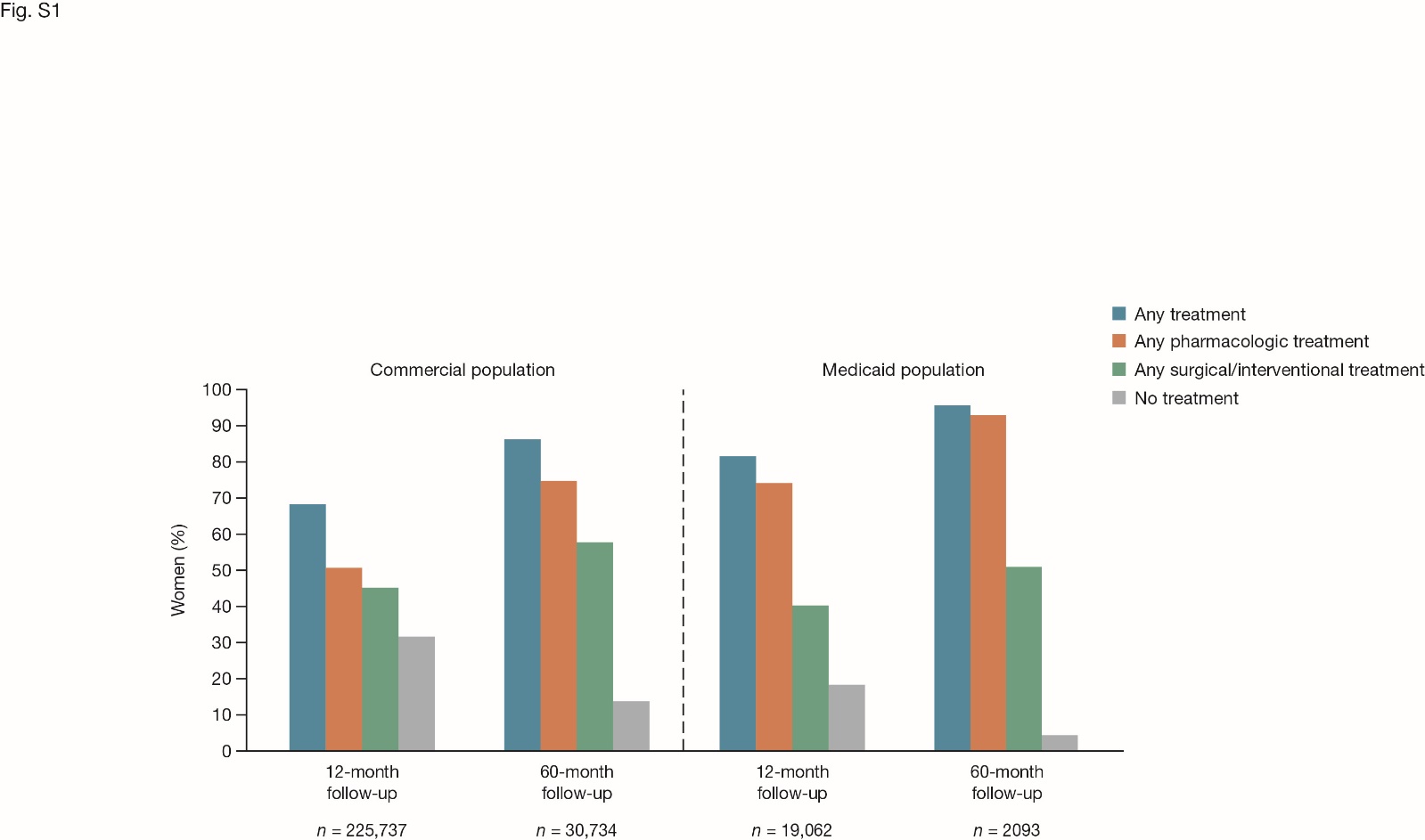

Supplement: Supplementary file 4 — Additional file 4: Figure S1. Proportion of women receiving any treatment over the 60-month follow-up for Commercial and Medicaid populations. [file 12905_2020_1005_MOESM4_ESM.docx]
